# Supplementary material for: Assessment of Morpho-Physiological, Biochemical and Antioxidant Responses of Tomato Landraces to Salinity Stress
Source: Plants (Basel). 2021 Apr 5;10(4):696. doi: 10.3390/plants10040696 (PMC8065849; doi:10.3390/plants10040696)
Supplement: Supplementary file 1 [file plants-10-00696-s001.pdf]

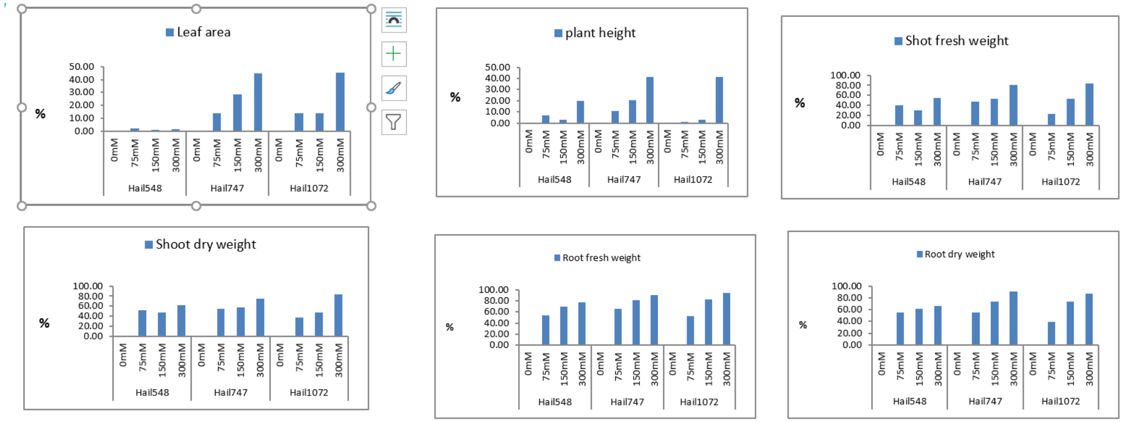

Figure S1. Percentage of reduction in leaf area, plant height, shoot and roots fresh and dry weights for the three Landraces at all NaCl treatments. Comparisons of means for the same level of landraces at Alpha 0.05, Standard error for: leaf area = 0.37, plant height = 0.36, shoot fresh weight = 0.74, shoot dry weight = 0.74, root fresh weight = 0.87, root dry weight = 0.68. Comparisons of means for different levels of landraces at Alpha 0.05, Standard error for: leaf area = 0.38, plant height = 0.32, shoot fresh weight = 0.65, shoot dry weight = 0.66, root fresh weight = 0.75, root dry weight = 0.68.

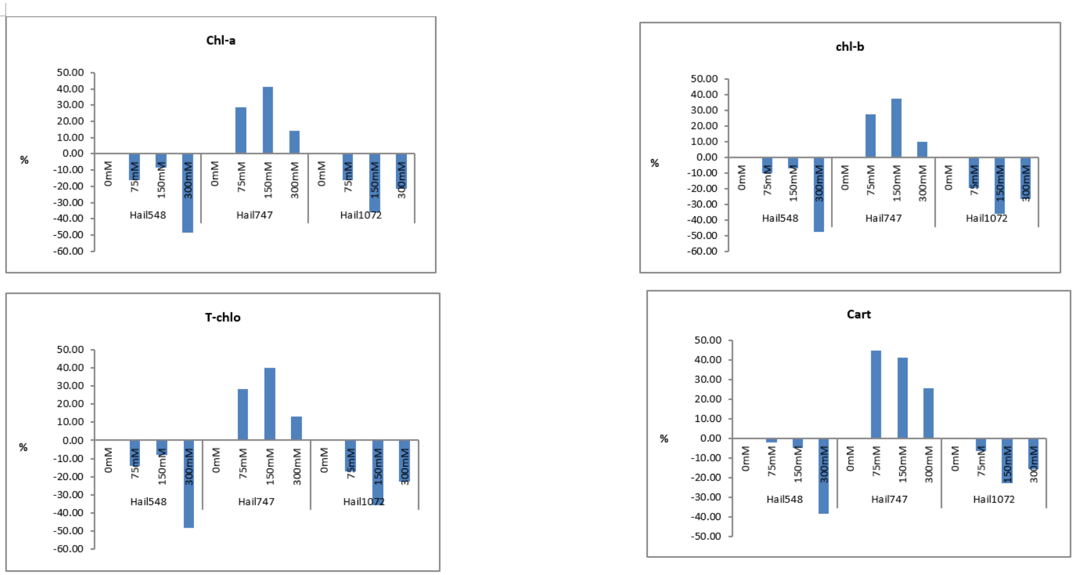

Figure S2. Reduction or increasing in chlorophyll _a and chlorophyll _b, total chlorophyll and carotene percentage for the three Landraces at all NaCl treatments. Comparisons of means for the same level of landraces at Alpha 0.05, Standard error for: Chlorophyll-a = 0.35, Chlorophyll-b = 0.53, total chlorophyll = 0.44, carotenoids = 0.32. Comparisons of means for different levels of landraces at Alpha 0.05, Standard error for: Chlorophyll-a = 0.62, Chlorophyll-b = 0.69, total chlorophyll = 0.66, carotenoids = 0.51.

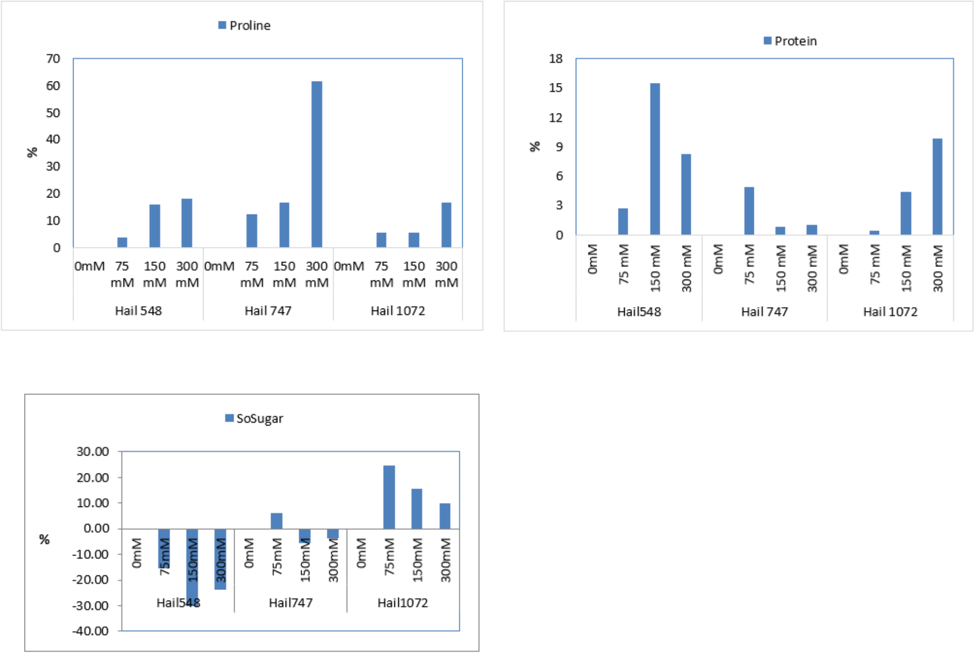

Figure S3. Increasing in proline and reduction in protein and behavior of soluble sugar percentage of percentage for the three Landraces at all NaCl treatments. Comparisons of means for the same level of landraces at Alpha 0.05, Standard error for: proline = 3.60, protein-b = 0.30, soluble sugars = 0.25. Comparisons of means for different levels of landraces at Alpha 0.05, Standard error for: proline = 5.0, protein-b = 0.33, soluble sugars = 0.42.

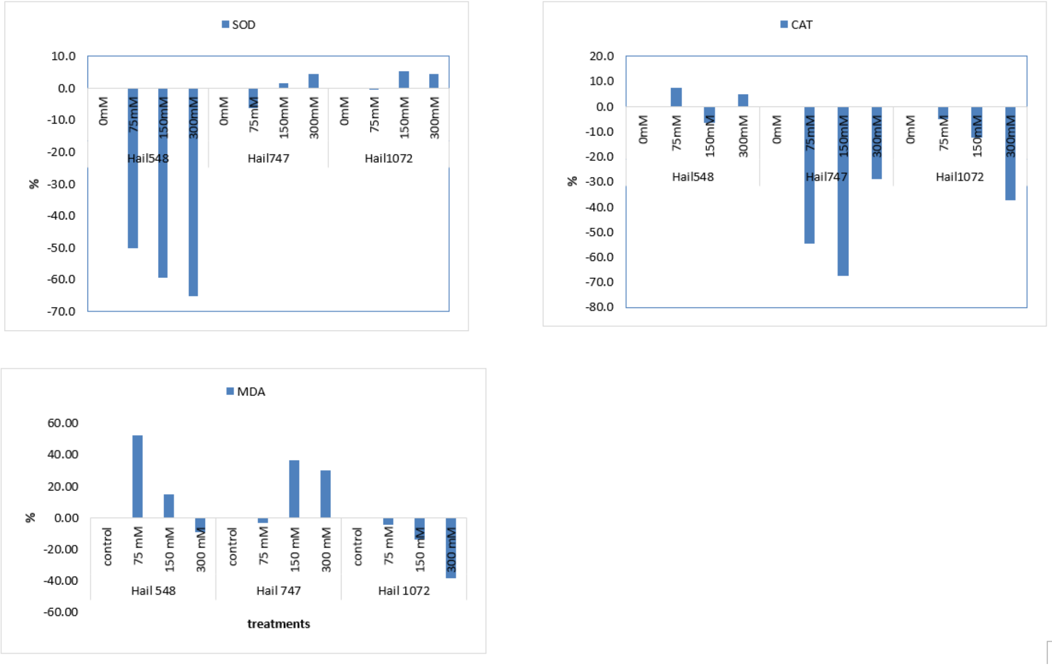

Figure S4. Reduction or increasing in SOD, CAT and MDA percentage for the three Landraces at different NaCl treatments. Comparisons of means for the same level of landraces at Alpha 0.05, Standard error for: SOD = 0.46, CAT = 0.49, MDA = 0.67. Comparisons of means for different levels of landraces at Alpha 0.05, Standard error for: SOD = 0.75, CAT = 0.59, MDA = 0.85.

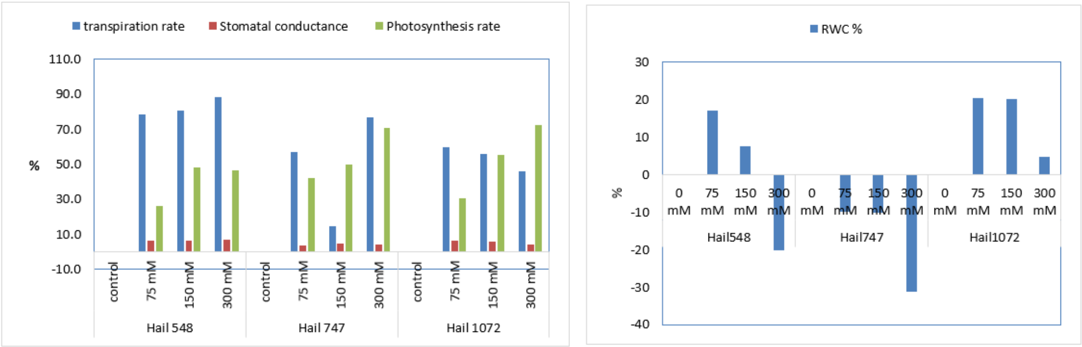

Figure S5. Reduction in transpiration rate, stomatal conductance and photosynthetic rate percentage and behavior relative water content (RWC) for the three Landraces at different NaCl treatments. Comparisons of means for the same level of landraces at Alpha 0.05, Standard error for: transpiration rate = 1.35, stomatal conductance = 0.10, photosynthetic rate = 1.0, relative water content = 1.6. Comparisons of means for different levels of landraces at Alpha 0.05, Standard error for: transpiration rate = 1.2, stomatal conductance = 0.10, photosynthetic rate = 0.90, relative water content = 2.2.

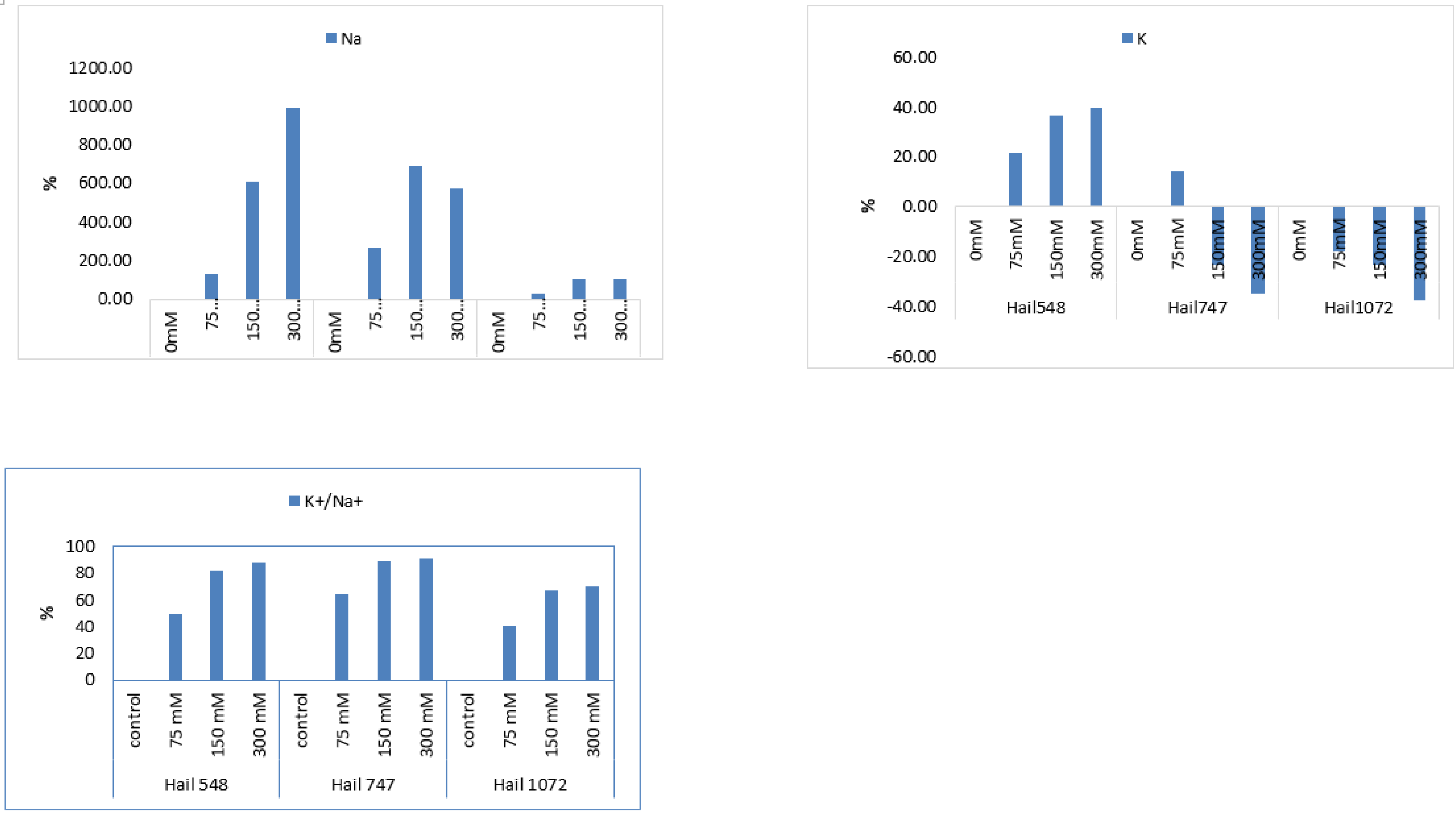

Figure S6. Increasing percentage in Na^+ and reduction or increasing percentage in K^+ and the behavior of potassium /sodium (K^+/Na^+) ratio for the three Landraces at different NaCl treatments. Comparisons of means for the same level of landraces at Alpha 0.05, Standard error for: $Na^+ = 4.35$, $K^+ = 0.30$, $K^+/Na^+ = 8.5$. Comparisons of means for different levels of landraces at Alpha 0.05, Standard error for: $Na^+ = 4.7$, $K^+ = 0.40$, $K^+/Na^+ = 9.7$.
